# Neuro-ophthalmology in the United Kingdom: providing a sustainable, safe and high-quality service for the future

**DOI:** 10.1038/s41433-024-03141-x

**Published:** 2024-05-22

**Authors:** Susan P. Mollan, Vaishnavi Menon, Alan Cunningham, Gordon T. Plant, Luke Bennetto, Sui H. Wong, Margaret Dayan

**Affiliations:** 1 Birmingham Neuro-Ophthalmology, University Hospitals Birmingham NHS Foundation Trust, B15 2GW, Birmingham, UK; 2 Translational Brain Science, Institute of Metabolism and Systems Research, University of Birmingham, Birmingham, B15 2TT UK; 3 Newcastle Eye Centre, Royal Victoria Infirmary, Queen Victoria Road, Newcastle upon Tyne, NE1 4LP UK; 4 University College London, London, UK; 5 Department of Neurology, Southmead Hospital, North Bristol NHS Trust, Bristol, BS10 5NB UK; 6 Moorfields Eye Hospital NHS Foundation Trust, City Road, London, UK; 7 Guys & St Thomas’ NHS Foundation Trust, London, UK

**Keywords:** Health care economics, Health occupations

Neuro-ophthalmologists play a major role in protecting vision and life [1–4]. Misdiagnosis prior to neuro-ophthalmology review may occur in up to 69% of cases [1] with a quarter coming to harm [2] due to inadequate history, examination, differential diagnosis, incorrect targeting and interpretation of investigations [2]. Studies also found that Neuro-ophthalmology review impacted on care in 99% and saved life or vision in 2% [2] however over one third of patients had a delay in their care [3]. These results suggest inadequacy of both access to neuro-ophthalmology services [5], and exposure to neuro-ophthalmology during general training.

“Neurophobia” was coined to reflect fear or rejection of clinical neurology by medical students [6], and emergency care trainees expressed greatest insecurity for neuro-ophthalmology out of all neurological disciplines [7]. This suggests more confidence and support is needed in recognising and managing neuro-ophthalmic conditions. Here, we report a survey of providers of neuro-ophthalmology care in the United Kingdom (UK).

An electronic survey was sent out to 256 United Kingdom Neuro-Ophthalmology Society (UKNOS) and British Isles Neuro-ophthalmology Club (BINOC) members (ophthalmologists and neurologists) between July and September 2021. There is some overlap in these memberships. None of the questions were mandatory. The data analysis used descriptive statistics.

Fifty three people responded to the survey (overall response rate of 35%). Twenty-three percent worked in a department with one neuro-ophthalmology consultant while 47% worked in a department with two or more. Only one-third worked in departments with neuro-ophthalmology fellows and only a quarter in a department with specialist trainees. Only three people worked with staff and associated specialist grade doctors on the neuro-ophthalmology team.

The majority (67%) worked with specially trained allied professionals who were brought in to cope with limitations in the medical workforce - mainly orthoptists but also optometrists and clinical nurse specialists. Over half of respondents had an unfilled medical post on their team - one-third consultant and one fifth fellowship positions.

Sixty three percent were fellows of the Royal Colleges of Ophthalmologists (FRCOphth) or Surgeons, 46% held membership or fellowship of the Royal College of Physicians (FRCP/MRCP), some having affiliations with both colleges, and a minority held a PhD. Neuro-ophthalmology training was by fellowship in 46%, trainee-selected component in 39%, observership in 37% with some having a combination. They voiced a strong opinion that Neuro-ophthalmologists should hold either FRCOphth or MRCP and certificate of completion of training (CCT) in medical ophthalmology, neurology or ophthalmology. A non-UK-based fellowship was cited as a desirable training opportunity.

Secondary care specialists were the main referrers (Fig. 1A). 72% of respondents felt they received too many referrals to deliver a high-quality service. Institutional factors preventing this were common and multifactorial: challenges in clinic booking, and non-availability of clinical notes (Fig. 1B). Access to diagnostics or onward referrals were also cited as problematic (Fig. 1B). One person reported no challenges to providing a high quality service! Only 26% neuro-ophthalmologists had dedicated urgent clinic slots, and 21% had access to dedicated urgent clinics. For 40%, overbooking clinics was the only method to see emergencies.

**Fig. 1:**
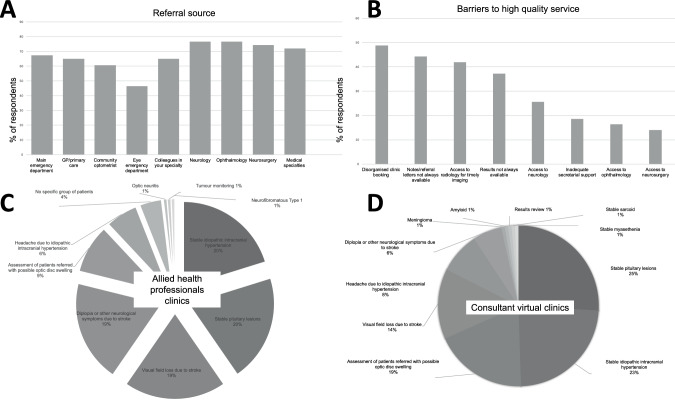
The results of the survey. **A** Portion of referring specialists to the UK neuro-ophthalmology services; **B** Barriers, in the opinions of current neuro-ophthalmologists, to providing a high quality neuro-ophthalmology service; **C** Virtual clinic diseases areas provided by consultants and **D** Out-patient clinics provided by allied health professionals focusing on different disease areas.

Allied health professionals ran their own disease-specific clinics (Fig. 1C). The majority of respondents (63%) ran virtual clinics with a variety of disease-specific themes (Fig. 1D). Most respondents worked with clinical protocols for conditions such as idiopathic intracranial hypertension (60.9%), pituitary lesions (54.3%), stroke (50%), optic neuritis (50%), non-arteritic anterior ischaemic optic neuropathy (26.1%) and acute onset double vision (34.8%). Multidisciplinary team meetings were part of a regular job plan for 78% of respondents.

This survey has provided unique insights to UK Neuro-ophthalmology practice with a high reliance in allied health professionals and cites barriers that impede delivery of high-quality services.

Only 75% worked in a department with specialist trainees resulting in a worrying lack of exposure to Neuro-ophthalmology for ophthalmology and neurology trainees. This will perpetuate ‘neurophobia’ potentially leading to misdiagnosis and harm and removes the opportunity to attract trainees into this highly rewarding specialty. With so many unfilled consultant and fellowship posts and 26% of UK ophthalmologists planning to leave the workforce over the next 5 years [8], the damage to the Neuro-ophthalmology workforce pipeline needs to be urgently fixed.

Neuro-ophthalmology is one of the 12 special interest areas in the new ophthalmology curriculum, which could help. However, it is not recognised as a subspecialty in the new neurology curriculum. Physicians trained in Neurology have contributed greatly to the practice and advancement of UK Neuro-Ophthalmology for more than a century. As a result of the decision not to include the specialty in core training will lead to further deskilling of all consultant Neurologists with respect to disorders affecting vision. Furthermore, only the availability of post CCT fellowships for Neurology trainees, which will need to be provided in Ophthalmology departments, could provide an opportunity for them to take up the specialty as consultants.

This survey suggests current service delivery for urgent cases is unplanned, chaotic and unsustainable. Solutions need to be found. Nationally the “re-development” of general ophthalmology and neurology services is required for cases that could be managed by an appropriate generalist. Regionally and locally investigational and robust clerical support is required. While innovation and service design are part of the solution to meet capacity, overall, more people need to be trained to deliver neuro-ophthalmology care. These issues are not unique to the UK [9–13]. However some of the driving factors may be different [14].

Due to the nature of this survey demographic information was not collected which could have informed on whether gender, age or racial disparities exist within the workforce. With only a 35% response rate, the results of the survey may not be generalisable. Another key limitation is the lack of a UK register of neuro-ophthalmologists. Therefore, using neuro-ophthalmology membership lists were deemed to be the most representative way to survey specialists.

We remain deeply concerned about the rising reports of misdiagnosis and harm in Neuro-ophthalmology patients [1, 2]. Competency in the appropriate methods of clinical examination and investigation is mandatory. We wish to work with our colleagues to provide robust training and encourage people to become neuro-ophthalmologists; and above all provide safe innovative models to meet the rising demand for neuro-ophthalmology services.

## Data Availability

Data are available upon reasonable requests to the corresponding author.
